# On the Environmental Factors Affecting the Structural and Cytotoxic Properties of IAPP Peptides

**DOI:** 10.1155/2015/918573

**Published:** 2015-10-25

**Authors:** Marianna Flora Tomasello, Alessandro Sinopoli, Giuseppe Pappalardo

**Affiliations:** ^1^CNR Institute of Biostructures and Bioimaging, Via P. Gaifami 18, 95126 Catania, Italy; ^2^International PhD Program in Translational Biomedicine, University of Catania, Viale A. Doria 6, 95125 Catania, Italy

## Abstract

Pancreatic islets in type 2 diabetes mellitus (T2DM) patients are characterized by reduced *β*-cells mass and diffuse extracellular amyloidosis. Amyloid deposition involves the islet amyloid polypeptide (IAPP), a neuropancreatic hormone cosecreted with insulin by *β*-cells. IAPP is physiologically involved in glucose homeostasis, but it may turn toxic to *β*-cells owing to its tendency to misfold giving rise to oligomers and fibrils. The process by which the unfolded IAPP starts to self-assemble and the overall factors promoting this conversion are poorly understood. Other open questions are related to the nature of the IAPP toxic species and how exactly *β*-cells die. Over the last decades, there has been growing consensus about the notion that early molecular assemblies, notably small hIAPP oligomers, are the culprit of *β*-cells decline. Numerous environmental factors might affect the conformational, aggregation, and cytotoxic properties of IAPP. Herein we review recent progress in the field, focusing on the influences that membranes, pH, and metal ions may have on the conformational conversion and cytotoxicity of full-length IAPP as well as peptide fragments thereof. Current theories proposed for the mechanisms of toxicity will be also summarized together with an outline of the underlying molecular links between IAPP and amyloid beta (A*β*) misfolding.

## 1. Introduction

Diabetes is a complex metabolic disorder characterized by chronic hyperglycemia and associated with macrovascular and microvascular complications [1]. It is estimated that the total number of people with diabetes will rise from 171 million in 2000 to 366 million in 2030 [2]. The two major forms of diabetes, classified by aetiology, are type 1 diabetes, which is characterized by an absolute deficiency in insulin resulting from autoimmune destruction of pancreatic *β*-cells, and type 2 diabetes which features combinations of decreased insulin secretion and insulin resistance. Type 2 diabetes mellitus (T2DM) accounts for about 90% of all the diabetes cases [1], and amyloid deposits are present in the pancreas of almost 90% of T2DM patients [3–5]. These amyloid deposits are primarily composed of a protein called the islet amyloid polypeptide (IAPP) or amylin [6]. The strong correlation between amyloid fibril formation and *β*-cell death indicates the possible contribution of islet amyloidosis in the increase of *β*-cell failure in T2DM. IAPP is a member of the calcitonin like family of polypeptide and it is synthesized as an 89-residue preprohormone. Removal of the 22-residue signal sequence leads to the 67-residue pro-IAPP, which is then processed in the Golgi and in the insulin secretory granule to give the 37-residue mature hormone [7, 8]. Additional posttranslational modifications include formation of an intramolecular disulfide bridge between residues 2 and 7 and amidation of the C-terminus. Mature IAPP is stored within the insulin secretory granules at a ratio of 1 : 50 to 1 : 100 relative to insulin and is cosecreted with insulin [9].

The physiological roles of IAPP are receptor mediated. The IAPP receptor is formed by a complex of the calcitonin receptor with a receptor activity modifying protein (RAMP) [10, 11]. IAPP binds the calcitonin receptor (CTR) in the absence of RAMPs, but the affinity is rather low.

The affinity of IAPP for the CTR-RAMP complex is higher, with an IC50 reported to be in the order of 8 nM for the CTR-RAMP-1 complex [12].

Circulating concentrations of IAPP have been reported to range between 3 and 5 picomolars in rats, rising to 15–20 picomolars with increased blood glucose levels [13]. However these values are unlikely to be relevant to amyloid formation since IAPP is stored at a much higher level in the insulin secretory granule, between 500 micromolars and several millimolars. This implies that the local concentration of IAPP after being released from the granule will be temporarily much higher than the circulating concentration. The normal physiological roles of IAPP are not completely understood in humans, but studies in rodent models show that IAPP is involved in the suppression of satiety and adiposity, as well as the regulation of glucose homeostasis, gastric emptying, suppression of glucagon release, vasodilatation, and the excretion of calcium, potassium, and sodium [13–21]. IAPP's effect appears to take place mainly within the area postrema (AP) in the CNS. Several works provide a critical examination of the physiological roles of IAPP [13–15, 20–22] but this will not be discussed in this review.

IAPP is phylogenetically well preserved and found in all mammals, but not all species form amyloids [23–25]. Mice and rats do not develop islet amyloids, but cats, nonhuman primates, and humans do. The human polypeptide is amyloidogenic* in vitro*, while the rat IAPP (rIAPP) is not, even though the two polypeptides differ at only six positions located in the region 18–29. Notably, rIAPP contains three proline residues within the 20–29 sequence and these are believed to be responsible for its inability to form amyloid. Much attention has been focused on the sequence within the 20–29 region and the role it plays in controlling amyloid formation. This portion of the polypeptide chain is considered to be one of the major determinants of the ability of IAPP variants to form amyloid [26–28].

While there is a strong correlation between the primary sequence of the 20–29 segment and* in vitro* amyloidogenicity, mutations outside of this region can eliminate amyloid formation, indicating that it cannot be the sole factor controlling IAPP's amyloidogenicity [29–31]. Moreover there is recent evidence that the nonamyloidogenic rIAPP peptide is endowed with toxicity toward *β*-cell cultures as well [32, 33].

In the present review we summarize recent progress in the field focusing on the influences of membranes, pH, and metal ions on the aggregation and cytotoxicity of IAPP and related peptide fragments. The current theories proposed for the mechanisms of toxicity will be also illustrated together with a brief account of the molecular links between hIAPP and amyloid beta (A*β*) misfolding.

## 2. Environmental Conditions Affecting IAPP's Aggregation Process

### 2.1. Amyloidogenic Properties of hIAPP

The connection between amyloidogenic proteins and disease was initially attributed to the amyloid form of the protein; however, increasing evidence suggests that the toxicity is due to intermediates generated during the assembly of amyloid fibers. hIAPP amyloid deposits have a characteristic structure shared with other aggregates that commonly occur in Alzheimer's, Huntington's, Parkinson's, and other degenerative diseases. These amyloid aggregates are defined by their primarily *β*-sheet structure and their supramolecular organization into long fibers with the protein backbone orthogonal to the fiber axis [5].

Besides these structural characteristics, amyloids share common physical properties such as the general mechanism of assembly despite the lack of sequence similarity between the individual proteins. Amyloids are formed through a nucleation dependent process. This leads to characteristic sigmoidal type kinetics in which fibril formation is minimal (the lag-phase) until a critical concentration of nuclei (oligomers) is reached, at which point fibrils growth proceeds exponentially. The kinetic of assembly is typically intricate and involves a variety of on- and off-pathway intermediates found among all amyloidogenic proteins [34, and references cited therein]. Different aggregation profiles can be observed when solutions of fibrillogenic peptides contain prenucleated seeds of aggregates.* In vitro*, the seeding of amyloid proteins solutions with preformed fibrils leads to dramatically faster fibril growth [35]. In fact when seeds are present or added to hIAPP preparations the kinetic profiles of aggregation show extremely short lag phases.

hIAPP fibrils are stable structures not easily degraded by proteases. Generally harsh conditions, such as the use of concentrated formic acid or organic solvents, are needed to obtain their depolymerization [36]. The stability of amyloid fibrils makes them easy to handle and has prompted scientists to study them.

On the contrary, the study of the monomeric and oligomeric structures of hIAPP in physiological buffers has been troubled by their instability and fast “spontaneous” aggregation of the species. The most recent and most detailed model for the structure of hIAPP fibrils suggests that they are composed of stacked layers of two symmetric hIAPP molecules. These molecules form a parallel *β*-sheet structure running perpendicular to the length axis of the fibril [37]. Residues 8–17 and 28–37 form the *β*-sheet structure with a loop region located between them, whereas residues 1–7 are largely unstructured. Another model of the hIAPP fibril suggests that the fibril is composed of three protofilaments, each based on stacking of single hIAPP molecules in which residues 9–37 participate in a planar *S* shaped fold forming the *β*-sheet structure [38, 39].

### 2.2. Interaction with Membranes and Membrane Mimicking Environment

Substantial evidence indicates that the membrane-mediated aggregation and subsequent deposition of hIAPP are linked to the dysfunction and death of pancreatic *β*-cells, but the molecular mechanisms of hIAPP deposition are poorly understood.

Many* in vitro* studies have shown that early oligomers of hIAPP formed during the aggregation process are the principal cytotoxic species and their toxicity is exerted through interactions with cell membranes. However many different hypotheses have been reported [39, 40].

Two general theories exploring membrane disruption by hIAPP or other amyloid proteins have been proposed to date, even though the mechanisms are not exclusive and both may be operative at the same time. The first, known as the “pore theory,” suggests that amyloid proteins form discrete pores within the membrane, either through a traditional ion channel type (“barrel stave”) or in localized ruptures of the membrane that resemble discrete pores in some respects [34, 41].

In fact hIAPP has been found to increase the conductance of planar phospholipid bilayers [40]. The second theory suggests that the cause of membrane permeabilization and cytotoxicity might be not a specific (oligomeric) hIAPP species, but rather the process of hIAPP fibril growth at the membrane [39]. It was found that the assembly of hIAPP fibrils at the membrane causes membrane disruption, possibly by forcing the curvature of the bilayer to unfavourable angles or by the uptake of lipids by hIAPP fibrils during fibril elongation at the membrane [39].

*β*-cells contain 2.5–13.2 mol% of anionic lipids and raft components (cholesterol and sphingomyelin) [40–46]. Thus, studies using model membrane surfaces mimicking the* in vivo* environment are useful for the understanding of amyloid deposition by hIAPP and for the development of effective therapeutically strategies [47]. Recent studies have demonstrated that cholesterol and lipid rafts content may affect the membrane driven aggregation of hIAPP and its accumulation on them [40–49]. Interestingly some lipids, particularly raft components, have been shown to be incorporated into amyloid deposits extracted from patients tissues affected with various amyloid diseases, including T2DM [40, 50]. The presence of phospholipid bilayers can reduce the lag-phase of hIAPP fibril formation, an effect that is most pronounced with negatively charged lipids. Since hIAPP has several positively charged residues, all of which are located at the N-terminal side of the peptide, it has been thought that this region might be involved in the initial interaction of hIAPP with lipids, namely, with negatively charged lipids. In fact it has been shown that membrane binding of hIAPP is most efficient when bilayer lipids are negatively charged [39].

The N-terminal part of hIAPP, whilst not significantly involved in hIAPP fibril growth, was proposed to be important in the light of hIAPP membrane interactions [51–53]. However, we found that the hIAPP 17–29 peptide, lacking the N-terminal part of hIAPP, was cytotoxic and was internalized in the mitochondrial compartment of RIN-5M cells [33]. Finally, similar to hIAPP, rIAPP can cause membranes to become permeable and cause cell toxicity, but it does not form amyloid [33, 54].

It is suggested that a transient membrane-bound *α*-helical structure of hIAPP might play an important role in IAPP fibrillation* in vivo*. This is probably because of the exposure of the 20–29 region to an aqueous environment [55, 56], even if it has been shown that the region 20–29 can interact with membrane models without affecting their integrity [57]. The exposure of this amyloidogenic region facilitates amyloid nucleation by enhancing the orientation and increasing local concentration of the peptide. This suggests that a complex interplay between the helical intermediates and membranes is involved in the membrane-mediated aggregation of hIAPP and influenced by the peptide concentration and lipid composition [39, 40, 58, 59].

Support to the importance of the helix intermediate on IAPP aggregations is given by the effect of helix-inducing solvent HFIP on aggregation. A low percentage of HFIP (1%) has a strong catalytic effect on aggregation while higher percentages trap the protein in a helical monomeric state [34, 39].

Since low percentage of HFIP affects the aggregation process it is important prior to starting any biophysical or biological measurements to exclude any trace of HFIP from the sample solution.

For these reasons it becomes important to adopt suitable procedures for sample preparation in order to achieve reproducible results [33].

### 2.3. pH Influence on the Conformational Polymorphism of IAPP

Fibrillization involves the conversion of soluble random coil, *α*-helical or *β*-sheet conformations into insoluble, aggregated *β*-pleated sheet structures. The conformational changes of IAPP are high conditional and even slight changes in parameters, such as pH, peptide concentration, presence of preformed seeds, or metal ions concentration, may lead to different forms of aggregates that can be either fibrillar or amorphous.

As reported above, hIAPP is stored at extremely high concentrations in the secretory granules of pancreatic *β*-cells [31], together with insulin and in the presence of high zinc concentration and an acidic environment (pH of 5.5). These conditions all together inhibit hIAPP aggregation and protect cells [60–63].

Conversely, the physiological pH of 7.4 for the extracellular matrix, into which amylin is secreted as the mature active hormone, favors fibril formation [63].

Amylin is a relatively simple system for the study of the effects of pH on fibrillization, because it has only two ionizable groups near neutrality, the *α*-amino group at the N-terminus and His18, because the C-terminus is naturally amidated in the mature hormone and it does not titrate with pH [62, 63].

It has been shown that protonation of the *α*-amino group leads to a relatively modest inhibition of fibrillization; this slight effect of the *α*-amino group is consistent with its location in a disordered part of the amylin fibril structure model [63]. Ionization of His18, which is part of the intermolecular *β*-sheet of amylin fibrils, causes a stronger inhibition of fibrillation as shown in different works. This is due to electrostatic repulsion between His18 stacked along the fibril axis [62, 63]. As a matter of fact, at higher pH the peptide readily aggregates to form amyloid [33, 64].

While the effect of pH on the morphology of the hIAPP aggregates has been reported by several groups [62, 63, 65, 66], the effect of pH on hIAPP toxicity cannot be determined directly in cell cultures and the only data so far available has been obtained by using H18R hIAPP mutants to simulate the effect of pH. Generally speaking, decreased cytotoxicity was observed when a permanently charged arginine is substituted for the histidine at position 18 in agreement with the amorphous nature of the IAPP aggregates formed at low pH values [62, 63].

Literature work on IAPP's aggregation process focuses also on biophysical studies on IAPP related shorter sequences, derived from fibrillogenic regions of the full length parent peptide. Among these, the hIAPP17–29 peptide has been proposed as a useful model able to reproduce some of the key features of the whole hIAPP1–37 [64, 67]. In particular the pH dependence of fibrillization was maintained by the hIAPP17–29 peptide because of the presence of the His residue that is the only aminoacid residue that can be titrated over the pH range that hIAPP can experience* in vivo* [62].

### 2.4. IAPP Interaction with Transition Metal Ions

Metals ions, notably Cu(II) and Zn(II), and metal ions dyshomeostasis have been linked to the progression of several pathologies, including Alzheimer's and T2DM diseases [68], as their presence has been found in amyloid deposits characteristic of the above mentioned pathologies. Cu(II) is known to interact with numerous proteins and enzymes and is required for normal cellular functioning [69]. However, uncomplexed Cu(II) may be very hazardous, particularly under reducing environments, such as the cytosolic compartment, where Cu(II) is readily converted into the potent and reactive Cu(I) ion known to produce tissue damaging ROS. Many plaque-forming proteins including *β*-amyloid and prions were found to interact with copper, affecting the redox cycle of the metal to generate ROS* in vitro* [70–72]. The presence of copper in amyloid deposits and the alterations in its levels observed in various biofluids and tissues of individuals suffering T2DM [73, 74] have promoted investigations on the effect of Cu(II) on hIAPP aggregation. Nonetheless the role played by the Cu(II)-hIAPP interaction in both aggregation and *β*-cell toxicity is still controversial. The majority of the studies dealing with the significance of Cu(II) and IAPP interactions emphasized the formation of H_2_O_2_ by human IAPP* in vitro*, the production of which is modestly elevated in the presence of Cu(II) [75, 76]. Remarkably, copper-dependent generation of H_2_O_2_ was found to directly contribute to the toxicity of the *β*-amyloid peptide in primary neuronal cell cultures [77]. A very recent paper reports on a clear interaction mechanism of Cu(II) and hIAPP in pancreatic *β*-cells as copper compounds increased hIAPP-induced cytotoxicity by facilitating the apoptotic effect of hIAPP. Furthermore, Cu(II) promoted ROS overproduction and mitochondrial disruption which might be the main reason for the enhanced apoptosis [78]. Previous studies on complex formation between Cu(II) and two other common amyloid proteins, that is, *β*-amyloid and prions, were consistent with these findings. It has been reported that, similar to human amylin, these two misfolded proteins form Cu(II) complexes with physiologically relevant affinities. Chelation of Cu(II) by these two amyloids greatly diminished the production of ROS by Cu(II) and reduced their toxicity, which further corroborates the hypothesis that hIAPP/Cu(II) complex may have a protective role in cells [79, 80]. Other authors reported that Cu(II) protects pancreatic *β*-cells from IAPP toxicity by increasing the activation energy needed for IAPP aggregation and by inhibiting IAPP-induced stress-kinase activation and signalling. Interestingly, the interaction of Cu(II) with hIAPP works as an antioxidant in cells rather than prooxidant activity as observed* in vitro*. This study further shows that both the hIAPP-induced ROS generation and the hIAPP-dependent activation of stress kinases (JNK) and caspases were blocked by preincubating hIAPP with Cu(II) [81].

Determinations of hIAPP fibrillization with ThT, AFM, and EM have revealed that Cu(II) inhibits the formation of mature hIAPP fibrils [82]. However, while some studies have shown that the copper-dependent decrease of hIAPP fibrillization was associated with the reduced hIAPP toxicity [81], others have pointed out that the copper-hIAPP interaction would increase hIAPP toxicity favouring the formation of toxic oligomers rather than inert fibrils. In fact, when supplied along with freshly dissolved hIAPP, Cu(II) decreased INS-1 rat insulinoma cell viability to a greater extent than what was observed in cells incubated with hIAPP alone [83]. In this case copper-induced oxidative stress was ruled out as the cause of toxicity, which was instead imputable to the inhibition of fibril formation and stabilization of toxic oligomeric forms. In fact it was demonstrated that Ni(II), which has similar binding preference as Cu(II) but forms non-redox-active complexes, also decreased both hIAPP fibril formation and increased hIAPP-related toxicity. Therefore, in the case of Cu(II), a decrease in amyloid formation does not correspond to a decrease in toxicity as Cu(II) possibly stabilizes either a toxic intermediate species of hIAPP or a toxic oligomerization-driven process.

Conformational changes can also explain the highest level of toxicity that has been observed after cell culture treatment with the hIAPP 17–29 fragment preincubated in the presence of Cu(II) and the absence of effects of copper on hIAPP1–37 spontaneous evolution towards toxic species [84]. Moreover, the observation that the rIAPP(17–29) peptide fragment can interact with copper(II) at physiological pH values, notwithstanding the lack of any common strongly coordination donor function, is noteworthy and imposes a revision of the rat amylin role [85].

The effect of Zn(II) on hIAPP fibrillogenesis and toxicity is of particular interest as *β*-cell granules contain millimolar concentrations of Zn(II), one of the highest levels in the body, and diabetics often exhibit zinc deficiency [5, 86, 87]. Zn(II) has a strong effect on hIAPP aggregation, although different from that one observed for A*β*; however, the physical basis of the effect is complex [88]. Zn(II) has generally an inhibitory effect on hIAPP aggregation since H18 is the only residue that changes the protonation state between pH 7.5 and pH 5.5. The pH dependence can be attributed to the protonation of H18, suggesting that the binding is localized near H18. NMR confirms this hypothesis by revealing that the greatest chemical shift perturbation accompanying binding of Zn(II) is localized near H18 [5, 68]. Since binding of Zn(II) to human amylin near H18 would result in electrostatic repulsion between adjacent *β*-sheets, this would disfavour the formation of mature fibrils [5, 89].

Another study shows that zinc binds and stabilizes prefibrillar aggregates of amylin during the lag-phase [60]. Zinc is involved in many aspects of glucose homeostasis, including paracrine regulation, insulin storage, and glucose stimulated cascades [90]. In *β*-cells zinc was proposed to be required for multiple steps in insulin synthesis crystallization and release [91, 92], but conclusive evidence is lacking. Interestingly mice lacking the only zinc transporter in *β*-cells, the ZnT8, were shown to become glucose intolerant when fed with a high fat diet [93].

## 3. Proposed Mechanisms and Pathways Involved in IAPP Cytotoxicity

Current studies performed in primates strongly support the concept that islet amyloidosis and *β*-cell apoptosis are two key determinants of islet dysfunction in T2DM [94–96]. The notion that IAPP evokes degeneration of pancreatic *β*-cells is nowadays acknowledged, but neither the precise nature of the harmful IAPP species nor the mechanisms of cytotoxicity have been clarified. Most studies carried out during the last decade have pointed out that hIAPP amyloid fibrils do not contribute directly to IAPP cytotoxicity, since there is no strong correlation between the extent of amyloid deposits and the disease severity [97]. A growing body of evidence has instead supported the toxic oligomer hypothesis which proposes that small membrane-permeant human amylin oligomers are the toxic form responsible for *β*-cell death. Konarkowska and colleagues found that mature hIAPP fibrils were not cytotoxic* in vitro*; rather the transition from random coil to *β*-sheet, coupled with de novo aggregation, was necessary for the induction of *β*-cells death [98]. In line with this concept, it was shown that rifampicin [99] which is able to prevent hIAPP fibrillization but not hIAPP oligomer formation [100] did not protect *β*-cells from apoptosis induced by either overexpression or application of hIAPP [101]. Similar amyloid aggregation and toxicity behaviours were also observed for other amyloidogenic peptides including amyloid-*β* linked to Alzheimer's disease and *α*-synuclein linked to Parkinson's disease [102, 103]. hIAPP toxic oligomers are present in *β*-cells of patients with T2DM and increase in abundance with obesity. Recently, antibodies which specifically recognized these assemblies, but not monomers or amyloid fibrils, have been exclusively identified in diabetic patients and have been shown to neutralize the apoptotic effect induced by these oligomers [104]. The presence of antibodies in sera of diabetic patients provides very strong evidence in support of the pathological relevance of the oligomers [105].

Another big unsolved issue is whether the hIAPP-induced apoptosis is triggered by species formed outside or inside cells. Based on studies carried out using cultured *β*-cells or transgenic animal model susceptible to hIAPP-evoked diabetes, *β*-cell apoptosis may be activated by hIAPP oligomers originating either on the surface of cells or within them [95, 97, 106–110]. IAPP oligomers in diabetic patients have been detected throughout the secretory pathway and are often associated with disrupted mitochondrial membranes [111] implying intracellular formation. Also oligomer immunoreactivity has been demonstrated by light microscopy in islets from hIAPP transgenic mice, but not in mice overexpressing comparable levels of nonamyloidogenic rodent IAPP or wild-type mice. Immunization against toxic oligomers did not protect these mice from developing diabetes, indirectly suggesting that toxic IAPP oligomers originate and act intracellularly [112, 113]. Recently, by using confocal microscopy, independent groups revealed that exogenous IAPP can enter the cell and this is associated with signs of cell death [32, 33]. Several mechanisms of toxicity, including receptor and nonreceptor-mediated events, have been proposed. A consensus in the field has been growing about the role of hIAPP interactions with cell membranes enabling disruption the cell membranes integrity, permeability, and functions, resulting in ions dishomeostasis, changes in signalling pathways, oxidative injury, and ultimately apoptotic cell death (for a detailed review see [114]).

### 3.1. IAPP-Induced Structural Alteration of Extra- and Intracellular Membranes

IAPP is natively unstructured but gains *α*-helical structure upon binding to anionic membranes and forms *β*-sheet-rich amyloid fibers during the progression of type II diabetes. Interaction between IAPP and cellular membranes was proposed as the cause of IAPP cytotoxicity and *β*-cell death in T2DM, since detailed ultrastructural investigations have revealed that islet amyloid was often in contact with *β*-cell membranes [115] and because these membranes presented concomitant morphological changes [116]. An important amount of data exists suggesting that toxic oligomers may be formed within membranes [42, 117–119]. Furthermore, the observation that toxic IAPP oligomers may escape from the secretory pathway, presumably by disrupting membranes of ER, Golgi, and secretory vesicles, is consistent with* in vitro* studies noting that toxic oligomers of amyloidogenic proteins induce membrane permeability and membrane disruption [42, 118–120]. It should be noted that since the combination of hIAPP and membranes in nondiabeticpeople does not normally result in *β*-cell death, particular T2DM-related surrounding conditions should exist that initiate hIAPP-induced membrane damage. An increase in the level of hIAPP, which is coproduced and cosecreted with insulin, in a state of insulin resistance, could initiate hIAPP oligomerization. More specifically, an altered ratio of insulin to hIAPP, as observed in diabetic patients [121], could lead to a decrease of the inhibitory effect of insulin on hIAPP amyloid fibril formation. However, the inhibition of insulin on hIAPP aggregation is only based on* in vitro* studies [122, 123]. On the other hand, a modified lipid composition of the *β*-cells, in particular an increase in negatively charged lipids, as inferred from studies with mouse and rat models for T2DM [124] could trigger an increase in hIAPP-membrane interactions.* In vitro* studies have shown that negatively charged lipids increase the rate of hIAPP fibril formation [125, 126] and also enhance hIAPP-induced membrane damage [127, 128]. The membrane itself could promote hIAPP growth by increasing the local concentration of (membrane bound) hIAPP and/or by promoting a specific orientation or conformation of the peptide that makes hIAPP molecules more susceptible to aggregation into oligomers or fibrils. Recent research shows that not only phospholipid bilayers, but also a polyanion like heparin [129] or a dichloromethane/water interface [130], can induce nucleation and aggregation of hIAPP. Finally glycosaminoglycans chains of the heparin sulfate proteoglycan (HSPG) Perlecan have been shown to significantly accelerate amyloid formation from IAPP and from the partially processed forms of pro-IAPP* in vitro* [131]. These results indicate that the overall charge of membrane, a hydrophobic/hydrophilic interface (both present in biological membranes), and some surrounding molecules are important factors that promote hIAPP fibril formation. In the case of the Alzheimer's disease-related A*β* peptide, it has been suggested that not a particular species but the ongoing amyloid fibrillogenesis is the true cause that is responsible for membrane damage [132]. Altogether, these notions suggest that a cytotoxic mechanism based on fibril growth at the membrane could represent a common mechanism for amyloid-induced cell death. Interactions of membrane-located hIAPP species with each other, or with hIAPP species in solution, lead to growth of fibrils at the membrane. The mechanism of membrane damage could entail growth of a rigid hIAPP fibril on a flexible phospholipids bilayer, which would result in a forced change in membrane curvature, deformation disruption, blebbing, and vesicle budding of cell membranes as observed in the presence of synthetic and cell-derived hIAPP [33, 116, 118].

A number of factors related to the formation of membrane-permeant oligomers might contribute to the dysfunction of pancreatic *β*-cells in T2DM in analogous of neurons in neurodegenerative diseases. Some of them will be discussed more in depth in the following sections.

### 3.2. IAPP-Induced Calcium Dyshomeostasis, ER Stress, and Autophagy

The delivery of toxic oligomers to the extra- or intracellular membranes might be expected to significantly affect signalling pathways involved in cell survival and cell death.

Calcium homeostasis and signalling not only is important for cell survival but also underpins the intracellular trafficking and regulated discharge of secretory and synaptic vesicles at the cell membrane. Remarkably, an increase in the intracellular Ca^2+^ concentration has been described as being provoked by amyloid oligomers in neurons and astrocytes [133, 134]. Notably Ca^2+^ concentration has been suggested to be an important cofactor able to modulate the mechanism of membrane destabilization, favouring one mechanism rather than another. In particular the presence of Ca^2+^ seems to enhance membrane disruption induced by hIAPP fibers through detergent like mechanism [135]. Yet, unregulated remodelling of the tubulin cytoskeleton consequent on hyperactivation of Ca^2+^ sensitive calpain might contribute to impaired intracellular vesicle trafficking [136], resulting in altered secretion of insulin and/or IAPP, exacerbating T2DM degenerations.

Several studies have reported that hIAPP overexpression or exposure induces defects in glucose stimulated insulin secretion (GSIS), possibly due to its capacity to aggregate and form intracellular oligomers [137, 138]. Zhu et al. [139] demonstrated that high concentrations of hIAPP inhibited the activity of voltage-gated calcium channels VCC, leading to a defect in the intracellular Ca^2+^ mobilization in pancreatic islets. They also suggested that this could explain the failure in GSIS. Soty and coworker overexpressed hIAPP in rat pancreatic islets using lentiviral infection, which allows long term hIAPP overexpression, and demonstrated that hIAPP overexpression altered the activity of the ATP-sensitive potassium channels (KATP), leading to defects in insulin and IAPP secretion in response to glucose. Moreover, authors reported an associated increase in ROS production and mitochondrial activity interpreted as a compensatory mechanism for counteracting these defects. Elevated ROS levels can, in turn, lead to the progressive loss of *β*-cell function therefore contributing to the further worsening of the T2DM pathological condition [140]. Other mechanisms described to account for IAPP-induced GSIS include the direct formation of Ca^2+^ permeable pores by the amyloid oligomers themselves and/or the activation of VCC [141] not only in the plasma membrane but also in the ER. ER stress has been proposed as being an important contributor to hIAPP-induced *β*-cell death since oligomeric hIAPP has been detected in ER membranes and exogenously added hIAPP has been reported to induce ER stress [111, 142]. However, the role of ER stress in hIAPP mediated toxicity* in vivo* is controversial, since ER stress is important in transgenic models that overexpress hIAPP at high levels but is not detected when lower levels of IAPP are produced in cultured islet [140–143]. Indeed, theoretically the ER is well defended from misfolding by the unfolded protein response, high concentrations of chaperon proteins, high Ca^2+^ concentration, and an oxidative environment. Mature insulin secretory vesicles should also be relatively protected from hIAPP aggregation because of the acidic pH and the high insulin concentration, both factors being known for their ability to inhibit IAPP aggregation [144, 145]. It should also be taken in consideration that in long-lived secretory cells, such as pancreatic *β*-cells, bearing a high burden of protein synthesis and folding, the disposal of misfolded or denatured proteins, and particularly those with a potential to form toxic oligomers, is essential. Therefore the detected increased toxic IAPP oligomers in T2DM may be due to defective oligomer removal [146]. The autophagy/lysosome system plays a key role in cellular adaptation to stress via clearance of misfolded proteins, damaged organelles, and/or oligomerization-prone proteins [147, 148] It has been reported that while proteasomal degradation is a critical part of the clearance of nonaggregate-prone proteins, intracellular accumulation of abnormal aggregated proteins in neurodegenerative diseases, such as amyloid plaque formation in Alzheimer's disease (AD), is associated with malfunction of autophagy [149]. Hence, autophagy seems to be more important for the removal of amyloidogenic hIAPP and disregulated autophagy may be more relevant to the pathogenesis of human T2DM rather than that of murine T2DM. In 2014 three independent groups have demonstrated that exposure of cultured *β*-cells to hIAPP increases autophagosome formation and that the inhibition of autophagy increases the vulnerability of *β*-cells to the cytotoxic effects of hIAPP. In mice with *β*-cells-specific expression of hIAPP, a deficiency in autophagy resulted in the development of overt diabetes, which was not observed in mice expressing hIAPP alone or lacking autophagy alone. Furthermore, lack of autophagy in hIAPP-expressing animals resulted in hIAPP oligomerization and amyloid accumulation in pancreatic islets, leading to increased *β*-cells death [150]. Also treatment of INS-1 cells with hIAPP enhanced cell death, inhibited cytoproliferation, and increased autophagosome formation [151].

### 3.3. IAPP-Induced Oxidative Stress and Mitochondrial Impairment

Oxidative stress, which is an imbalance between the generation of ROS and the cells antioxidant defence capacities provided by glutathione and other reducing molecules, has been associated with a number of diseases, including cancer, heart diseases, Alzheimer's disease (AD), and T2DM [152]. At high concentrations, ROS can cause significant damage to cell structures, nucleic acids, lipids, and proteins, leading to apoptotic or necrotic cell death. Growing support for the involvement of oxidative stress in islet dysfunction and death has also been discussed [153]. Interestingly, antioxidant treatments have been shown to be effective in reducing ROS levels and *β*-cells apoptosis, suggesting that ROS mediate in IAPP toxicity [33, 108, 154, 155]. Being the energetic core of the cell, mitochondria represents also the main source of oxygen radicals, and alteration in the bioenergetic equilibrium of cells often results in oxidative stress. Therefore, it is not surprising that both alteration in mitochondrial impairment and oxidative stress are associated to a given disorder. The disruption of mitochondrial membranes by proximate toxic oligomers, which escaped from the secretory pathway, also provides a mechanism for the well-recognized contribution of mitochondrial dysfunction to overall cellular dysfunction in both *β*-cells in T2DM and neurons in neurodegenerative diseases [106, 156–158]. By using quantitative proteomics, Lim and coworker [159] demonstrated that hIAPP gains toxic features affecting the oxidative phosphorylation (OXPHOS) system, which includes the electron transport system (complexes I to IV) and F_1_F_0_ATP synthase (complex V), the oxygen consumption (as measured by an altered respiratory states), and the ROS levels. This demonstrates that different regulatory systems of the cells, associated with mitochondrial function and metabolism, are deregulated by hIAPP emphasising a role for oxidative stress in T2DM. Accordingly, cryoimmunogold labelling has also been used to directly reveal that the toxic oligomers of IAPP form within the secretory pathway, escaping and disrupting cellular membranes including mitochondrial membranes [112, 113]. A recent hypothesis was made in order to explain the ability of IAPP to enter the cell. The clustering of positive charges from each IAPP peptide chain at the interface with membrane would result in the formation of membrane-bound *α*-helical aggregates endowed with cell penetrating features [32]. It has been recently reported that also hIAPP17–29 enters the cell despite possessing only the histidine residue as possible cationising site. As expected for cationic molecules, both fluorescent F-hIAPP17–29 and F-hIAPP1–37 can be taken up into the mitochondrial matrix. The presence of this cationic peptide in the matrix leads to mitochondrial depolarization, opening of the permeability transition pore (PTP), causing swelling and rupture of the outer mitochondrial membrane, thus resulting in the rapid release of the peptide in the cytoplasm. In fact, mitochondrial swelling leads to the leakage of a series of mitochondrial proapoptotic factors resulting in cell death [160]. Therefore these mitochondrial toxic peptides selectively target the respiratory complexes I and II causing oxidative phosphorylation deficits and consequently the impairment of ATP production, oxidative stress, and energy deficits, ultimately resulting in cell death induction [161]. Disruption of the lysosomal compartment may also affect the redox balances of the cell.

### 3.4. Involvement of Death Receptors and Kinases Signalling in IAPP-Induced Apoptosis

Several groups have described activation of multiple apoptosis pathways following hIAPP exposure, including the sequential activation of caspase-8 and caspase-3 and the Jun NH_2_-terminal kinase (JNK)-1/c-Jun and p38 kinase pathways [162]. Oligomeric hIAPP might interact with *β*-cell membranes in a specific manner that activates death receptor signaling to trigger apoptosis. Several death receptor-signalling/death receptor-inducing molecules, including Fas and Fas-associated death domain (FADD), have been implicated in *β*-cell destruction in various cellular and animal models of immune-mediated diabetes and type 1 diabetic individual. Increased *β*-cell Fas expression has also been reported in type 2 diabetic individuals [163]. Cell-surface death receptor Fas and the related death domain protein FADD were shown to be important contributors in the pathway by which hIAPP evokes *β*-cell apoptosis [164].

Numerous studies have also indicated that the c-Jun N-terminal kinase (JNK) pathway has also been reported as a critical mediator of *β*-cell apoptosis induced by both exogenous and endogenous hIAPP. Islet amyloid increased mRNA levels of both the extrinsic (Fas, Fadd) and the intrinsic (Bim) apoptotic pathways markers, caspase-3, and the antiapoptotic molecule Bclxl in a JNK-dependent manner [155].

## 4. The Nonfibrillogenic Rat IAPP Can Turn Toxic to ***β***-Cells

Unlike hIAPP, rat IAPP (rIAPP) is a nonamyloidogenic form that does not accumulate and does not induce oligomer and/or fibril deposition in *β*-cells [23–28]. However, a conspicuous body of evidence is emerging suggesting that nonfibrillogenic rodent IAPP species may also be harmful to *β*-cells. For instance, rIAPP was found that to produce comparable H_2_O_2_ concentrations, indicating that amylin aggregation and amylin-induced ROS are unrelated processes [83]. Remarkably, some authors demonstrated that the full-length rIAPP peptide and its short derivate rIAPP17–29 are cytotoxic to cultured RIN-M cells and this effect is reverted by previous treatment with antioxidants [33]. Similar experimental evidence about the rIAPP1–37 cytotoxicity was also reported by others [32, 165]. Yet, considering some recent papers dealing with rIAPP/membrane interaction [34, 166] together with the evidence that small aggregates can be detected also in rIAPP samples, an rIAPP-associated cytotoxicity could be conceivable. Very recent work attempted establishing the relationship between the observed cytotoxicity of rIAPP1–37 and rIAPP8–37 and their ability to form aggregated species. Authors observed that, in analogy to hIAPP, rIAPP toxicity could be inhibited by an amylin receptor antagonist (AC 187) and a caspase inhibitor (zVAD-fmk). In addition they did not rule out any possible contribution of rIAPP oligomeric species to the toxicity of their preparations [165]. Conversely, in keeping with the* in vitro* evidence that no aggregated forms could be detected for rIAPP by using CD and DLS experiments, *β*-cells treated with rIAPP1 were not reactive when immunostained with the A11 antibody, suggesting that oligomers formation is not a stringent prerequisite for the observed rIAPP cytotoxicity. At the moment the hypothesis which seems more appropriate is that rIAPP toxicity might relate to a different mechanism compared to hIAPP. As a matter of fact fluorescent rat IAPP was detected in the lysosomal/endosomal compartment, whereas fluorescent hIAPP was seen in mitochondria [33]. As Magzoub and Miranker hypothesized, rat IAPP can be endocytosed and accumulated into lysosomes where it reaches a critical concentration, resulting in organelle membrane disruption and cytotoxicity [32]. In line with this hypothesis, it was observed that prolonged incubation of the fluorescent rIAPP resulted in spreading of the fluorescent signal throughout the cytosol, thereby suggesting that the peptide has disrupted the lysosomal membrane. This event was associated with the condensation of nuclei and the appearance of other morphological alterations typical of apoptosis [33].

## 5. Molecular Links between T2DM and AD

A variety of interconnecting factors links T2DM with Alzheimer's disease AD [167–169]. This would be not surprising given that aging is a well-known common risk factor for both pathologies. From a conformational point of view hIAPP and A*β* are more than unrelated molecules: both can populate several conformational states ranging from random coil to *α*-helix or *β*-sheet structures depending on the environmental surroundings. Similar to A*β*, IAPP is mainly unstructured in the monomeric state, but it can readily adopt *α*-helical conformation in the presence of helix-inducing fluorinated alcohols (TFE and HFIP) [170] or both *α*-helical and *β*-sheet conformations in a membrane mimicking environment [171, 172]. Amyloid cytotoxicity by membrane damage has been suggested not only for hIAPP but also for A*β*, and induction of nonselective ion channels and/or disruption of membranes of the secretory pathway is also consistent with cytotoxicity mediated by increased cytosolic Ca^2+^ in AD [136, 173, 174]. Oligomerization/aggregation of A*β* and hIAPP that induce oxidative stress in neurons and *β*-cells, respectively, as well as elevated tissue copper levels found in the brain and pancreas of the subjects harbouring amyloid plaques, are also common features shared by T2DM and AD pathologies. Despite the absence of a large sequence homology between A*β* and hIAPP (about 25% sequence identity and 50% sequence similarity) both aggregated oligomeric forms of these peptides display a common conformation structure [175]. In both cases, the oligomer toxicity is inhibited by oligomeric-specific antibodies, which suggests a common mechanism of toxicity shared by the two fibrillogenic peptides [176]. Finally both A*β* and hIAPP are substrates for insulin degrading enzyme (IDE) a metalloprotease involved in the homeostasis of these two peptides in the brain [177–179].

Disturbance of Cu(II) and Zn(II) physiological levels is another factor joining T2DM and AD. Whereas A*β*-metal ion interaction has been extensively investigated, since the recognition of the fundamental role played by transition metal ions dyshomeostasis in the etiopathology of AD [180–184], less data is available from the literature concerning the role played by Cu(II) or Zn(II) in the IAPP-associated aggregation and toxicity in T2DM. In A*β*, metal ion interactions occur mainly within the 1–16 N-terminal region where a variety of metal coordinating functional groups, from the side chains of the His, Asp, or Glu aminoacid residues, in addition to the N-terminal free amino group, are present. By contrast in hIAPP the imidazole moiety of the histidyl residue at position 18 and the N-terminal amino group are the only physiologically relevant anchoring sites for metal coordination. A lower affinity for these metal ions is therefore expected for hIAPP in comparison with A*β*.

The similar pathological parallels between AD and T2DM have allowed recent hypotheses suggesting that AD might be considered as a neuroendocrine-like disorder, in other words a sort of “type 3 diabetes” [185]. Epidemiological studies indicate that the incidence of AD is 2 to 5 times higher in individuals with T2DM [186]. Insulin resistance and metabolic dysfunction related with impaired glycemia in T2DM have been related to cognitive decline in MCI cases [187]. Both T2DM and AD are characterized by insoluble *β*-sheet rich fibrillar aggregates of IAPP or A*β* peptides in the pancreas and brain, respectively. IAPP aggregation is associated with pancreatic *β*-cell loss, whereas clumps of A*β* lead to neuronal cell loss. The recent identification of deposits of amylin in the brains of people with Alzheimer's disease, as well as combined deposits of amylin and A*β* plaques, strengthens evidence on the relationship between T2DM and AD indicating also that amylin is a second amyloid and, potentially, a new biomarker for age-related dementia and Alzheimer's [188]. Finally, it is noteworthy that the monomeric form of A*β* seems to behave as a brain protective factor able to regulate synaptic activity and to activate insulin/IGF-1 receptor signalling. Thus it has been suggested that depletion of *β*-amyloid monomers, occurring in the preclinical phase of AD, might be the cause of early insulin/IGF-1 signalling disturbances that anticipate cognitive decline [189, 190].

## 6. Conclusions

According to major research, amylin or IAPP is a pancreatic peptide hormone naturally produced with insulin and appears to be involved in the cause of both main types of diabetes. Current efforts are focused on understanding the local environmental conditions and interactions with metal ion that can directly influence IAPP aggregation process. The molecular mechanisms underlying amyloid formation are not completely known and particularly their temporal sequence, the hierarchy, and the threshold dividing physiology from pathology are unknown.

That T2DM causes severe brain injury with time is already known, and although there has been a lot of speculation about why that occurs, no conclusive evidence has been found until now. Amylin can readily cross the blood brain barrier (BBB) and presumably interact with A*β* in the brain. There is clear evidence that amylin may be associated with A*β*, but the nature of their relationship remains unclear. In any case, the consciousness of such a high connection may support the idea that beneficial therapeutic strategies against T2DM might also be beneficial against AD. Thus research efforts at preventing oligomers and fibril formation by IAPP, together with a clear elucidation of the role played by metal ions in T2DM, might represent a new research field attempting to develop drugs as a cure for both pathologies [191–195].
